# [Retracted] Methylation and intratumoural heterogeneity of 14-3-3 σ in oral cancer

**DOI:** 10.3892/or.2026.9165

**Published:** 2026-07-20

**Authors:** Ujjal Kumar Bhawal, Keiichi Tsukinoki, Tomonori Sasahira, Fuyuki Sato, Yusuke Mori, Noriko Muto, Masaru Sugiyama, Hiroki Kuniyasu

Oncol Rep 18: 817–824, 2007; DOI: 10.3892/or.18.4.817

Subsequently to the publication of the above article, a concerned reader drew to the Editor's attention that the tubulin control blots featured in Fig. 1C on p. 819 were also apparently included as control blots in Fig. 5 of a paper published previously by the same research group in the journal *Oncology*, albeit the experimental conditions were reported to be different. In addition, concerning the western blots shown in Fig. 1C, there appeared to be the duplication of a pair of control protein bands where the respective lanes of the gel were labelled differently, and the bands for the T2 experiment were strikingly similar to those featured as the MKN45 bands, although turned through 180°, suggesting that this figure may have potentially undergone inappropriate editing.

After having evaluated these data independently in the Editorial Office, the concerns of the reader were shown to be well founded. Therefore, in view of the uncertainties regarding the assembly of the western blot data in Fig. 1C, the Editor of *Oncology Reports* has decided that this paper should be retracted from the Journal on account of a lack of confidence in the presented data. The authors were asked for an explanation to account for these concerns, but the Editorial Office did not receive a reply. The Editor apologizes to the readership for any inconvenience caused.

